# mTOR‐mediated autophagy in the hippocampus is involved in perioperative neurocognitive disorders in diabetic rats

**DOI:** 10.1111/cns.13762

**Published:** 2021-11-16

**Authors:** Xiaohui Chen, Fei Gao, Cuicui Lin, Andi Chen, Jianhui Deng, Pinzhong Chen, Mingxue Lin, Bingxin Xie, Yanling Liao, Cansheng Gong, Xiaochun Zheng

**Affiliations:** ^1^ Department of Anesthesiology Shengli Clinical Medical College of Fujian Medical University Fujian Provincial Hospital Fuzhou China; ^2^ Department of Anesthesiology Fuzhou Second Hospital Affiliated to Xiamen University Fuzhou China; ^3^ Fujian Provincial Institute of Emergency Medicine Fujian Provincial Key Laboratory of Emergency Medicine Fuzhou China

**Keywords:** autophagy, diabetes mellitus, hippocampus, mTOR, perioperative neurocognitive disorders

## Abstract

**Introduction:**

Perioperative neurocognitive disorders (PND) are common neurological complications after surgery. Diabetes mellitus (DM) has been reported to be an independent risk factor for PND, but little is known about its mechanism of action. Mammalian target of rapamycin (mTOR) signaling is crucial for neuronal growth, development, apoptosis, and autophagy, but the dysregulation of mTOR signaling leads to neurological disorders. The present study investigated whether rapamycin can attenuate PND by inhibiting mTOR and activating autophagy in diabetic rats.

**Methods:**

Male diabetic Sprague‐Dawley rats underwent tibial fracture surgery under isoflurane anesthesia to establish a PND model. Cognitive functions were examined using the Morris water maze test. The levels of phosphorylated mTOR (p‐mTOR), phosphorylated tau (p‐tau), autophagy‐related proteins (Beclin‐1, LC3), and apoptosis‐related proteins (Bax, Bcl‐2, cleaved caspase‐3) in the hippocampus were examined on postoperative days 3, 7, and 14 by Western blot. Hippocampal amyloid β (Aβ) levels were examined by immunohistochemistry.

**Results:**

The data showed that surgical trauma and/or DM impaired cognitive function, induced mTOR activation, and decreased Beclin‐1 levels and the LC3‐II/I ratio. The levels of Aβ and p‐tau and the hippocampal apoptotic responses were significantly higher in diabetic or surgery‐treated rats than in control rats and were further increased in diabetic rats subjected to surgery. Pretreatment of rats with rapamycin inhibited mTOR hyperactivation and restored autophagic function, effectively decreasing tau hyperphosphorylation, Aβ deposition, and apoptosis in the hippocampus. Furthermore, surgical trauma‐induced neurocognitive disorders were also reversed by pretreatment of diabetic rats with rapamycin.

**Conclusion:**

The results demonstrate that mTOR hyperactivation regulates autophagy, playing a critical role in the mechanism underlying PND, and reveal that the modulation of mTOR signaling could be a promising therapeutic strategy for PND in patients with diabetes.

## 1 INTRODUCTION

Perioperative neurocognitive disorders (PND) occur relatively frequently after surgery and are mainly characterized by the impairment of cognitive function, especially learning, memory, and attention.^1^, ^2^ Generally, patients recover from PND over time, but it may persist for weeks, months, or even years, result in prolonged hospitalization time, increase hospitalization costs, significantly delay postoperative recovery, and increase patient morbidity and mortality.^3^, ^4^ Diabetes mellitus (DM) is an increasingly serious global health issue affecting more than 431 million adults worldwide, and it is estimated that approximately 25% of diabetic patients will require surgery.^5^, ^6^ Recent evidence has suggested that DM is associated with mild‐to‐moderate cognitive dysfunction and is a major risk factor for dementia and Alzheimer's disease.^7^, ^8^, ^9^ A large number of clinical studies have revealed that patients with diabetes or hyperglycemia have a significantly higher incidence of PND than diabetes‐free persons and that these conditions might worsen perioperative neurological outcomes.^10^, ^11^, ^12^ Nevertheless, the pathogenesis and effective treatment for PND in diabetic patients remain largely unclear.

Autophagy is a cell survival pathway essential for cellular homeostasis that facilitates the recycling of obsolete cellular constituents and the elimination of damaged organelles and protein aggregates.^13^ Autophagy is partially responsible for eliminating amyloid β (Aβ) aggregates and neuronal tau,^14^ and defective autophagy responses have been demonstrated to cause Aβ accumulation and abnormal tau phosphorylation and to aggravate cognitive dysfunction in several neurodegenerative disorders.^15^, ^16^, ^17^ In addition, evidence has accumulated to report that upregulation of autophagy exerts crucial neuroprotective action in brain injury and neurodegenerative disorders.^18^, ^19^, ^20^ However, little is currently known about the relationship between autophagy and PND. It is now well established that diabetes is an independent risk factor for PND and is additionally connected with abnormal autophagy function. Several studies have shown that autophagic activity in the hippocampus is impaired, which contributes to cognitive impairment in diabetic rats.^21^, ^22^ In view of this, we hypothesized that autophagy might participate in PND and that enhanced autophagy could thus be useful for alleviating cognitive impairment, but the underlying mechanisms are still largely unknown.

Mammalian target of rapamycin (mTOR) is a highly conserved serine‐threonine kinase that is a master regulator of cell metabolism, proliferation, growth, and survival.^23^ In the central nervous system, the mTOR signaling pathway plays a critical role in modulating physiological functions, including synaptic plasticity, memory storage, and cognition.^24^ Evidence has identified that dysregulation of mTOR signaling is involved in cognitive impairment by modulating autophagy.^25^, ^26^ Kong et al.^27^ showed that liraglutide could alleviate diabetes‐induced hippocampal neuronal apoptosis and cognitive decline by promoting autophagy via mTOR signaling. Moreover, rapamycin, an mTOR inhibitor, has been shown to effectively reduce abnormal Aβ and tau protein deposition and improve learning and memory abilities by promoting autophagic flux in neurodegenerative diseases.^28^, ^29^ Therefore, it can be concluded that the modulation of autophagy via the mTOR pathway could be a promising novel strategy for the treatment of PND in diabetic patients.

In the present study, to test our hypothesis, we explored whether orthopedic surgery leads to the hyperactivation of mTOR signaling in the hippocampus of diabetic rats and whether inhibition of mTOR with rapamycin pretreatment was capable of improving autophagy impairment and cognitive dysfunction following surgery.

## 2 METHODS AND MATERIALS

### 2.1 Animals

Clean‐grade male Sprague‐Dawley rats weighing 200 ± 20 g were obtained from Fujian Medical University. The animals were housed in standard laboratory rat cages with five rats in each cage. The rats were maintained under a 12‐h light/dark cycle at 20–22°C with free access to food and water. A total of 648 male rats were used in the present study, and they were randomly allocated into different experimental groups.

### 2.2 Experimental protocol and animal models

#### 2.2.1 Experimental protocol 1

First, to investigate the effects of surgical trauma on the activation of mTOR, autophagy activity, hippocampal Aβ and phosphorylated tau (p‐tau) levels, and cognitive function in diabetic rats on days 3, 7, and 14 after surgery, male Sprague‐Dawley rats were divided into four groups: (1) sham‐operated control rats (Con group), (2) streptozotocin (STZ)‐induced diabetic rats undergoing sham surgery (DM group), (3) rats undergoing orthopedic surgery (Sur group), and (4) diabetic rats undergoing orthopedic surgery (DM + Sur group) (*n* = 4 per group).

#### 2.2.2 Experimental protocol 2

Moreover, to specifically determine whether hyperactivation of mTOR signaling contributed to PND via the modulation of autophagy in diabetic rats, the mTOR inhibitor rapamycin was applied in our study. Rapamycin was first dissolved in DMSO and then diluted in saline (final DMSO concentration, <1%). Then, male Sprague‐Dawley rats were further divided into eight groups: (1) control rats received vehicle (Veh group), (2) STZ‐induced diabetic rats (DM group), (3) diabetic rats treated with vehicle (DM + Veh group), (4) diabetic rats treated with rapamycin (DM + Rapa group), (5) rats undergoing orthopedic surgery and treated with vehicle (Sur + Veh group), (6) rats undergoing orthopedic surgery and treated with rapamycin (Sur + Rapa group), (7) diabetic rats undergoing orthopedic surgery and treated with vehicle (DM + Sur + Veh group), and (8) diabetic rats undergoing orthopedic surgery and treated with rapamycin (DM + Sur + Rapa group) (*n* = 4 per group). Rats in groups 4, 6, and 8 received intraperitoneal injections of 0.5 mg/kg/day rapamycin for 5 days prior to undergoing orthopedic surgery, with the final dose administered 2 h prior to surgery, and the other groups received the same volume of DMSO as the vehicle. The rapamycin dose, method of administration, and timing of administration were selected based on previous reports^30^

#### 2.2.3 Generation of STZ‐induced diabetic rats

A diabetic rat model was established by a single intraperitoneal injection of streptozotocin (STZ, 50 mg/kg, Sigma) as previously described.^31^ Seventy‐two hours after STZ injection, blood samples were collected from the tail vein to conduct a basal blood glucose level test. A blood glucose level >16.7 mM indicated successful establishment of the diabetic rat model.

#### 2.2.4 Establishment of the PND model

The PND rat model was established by performing internal fixation of tibial fractures under isoflurane anesthesia as previously described.^32^ Briefly, a middle incision was performed on the left tibial plateau, and then, a 20‐G pin was implanted into the tibial intramedullary canal from the hole drilled at the trochanter of the tibia. The periosteum was then stripped, osteotomy was performed at the junction of the middle and distal thirds of the tibia, and the incision was continuously sutured. The rat temperature was kept between 36°C and 37°C by a warming pad during the procedure. Ropivacaine (1%) was given subcutaneously to relieve postoperative pain.

### 2.3 Morris water maze test

The Morris water maze (MWM) test was performed to test the cognitive function of diabetic and nondiabetic rats on days 3, 7, and 14 after surgery as previously described.^33^ The maze (150 cm in diameter and 50 cm in depth) was divided into four quadrants, and a platform (12 cm in diameter) was submerged 1 cm below the water line in one quadrant of the pool. In the MWM test, a video camera connected to the computer running the tracking software (Panlab) was placed above the center of the maze to record and analyze *the* rats’ movements. During the place navigation test, the rats were randomly placed in the water facing the pool wall and allowed 60 s to find the escape platform. Rats that failed to find the submerged platform in time were guided to the platform, where they remained for 30 s. Rats received four trials per day for 5 consecutive days. The average escape latency time was measured to evaluate spatial learning and memory ability. On the sixth day, the probe trial was performed by removing the platform, and rats were allowed to swim freely for 90 s. The number of platform crossings and the time spent in the target quadrant were recorded to evaluate spatial memory ability. Furthermore, to exclude the possibility that any learning and memory impairments observed in the MWM test were caused by decreased rat mobility due to slower bone healing in the DM group, swim speed (cm/s) was also recorded. All the behavioral data, including the escape latency, swim speed, time spent exploring the target quadrant, and the number of platform crossings from each rat and trial, were collected by the tracking system connected to a video camera and were later analyzed by a trained observer blinded to the treatment groups.

### 2.4 Western blot analysis

Experiments were conducted as previously described.^33^ The harvested hippocampal tissues were homogenized using lysis buffer supplemented with protease and phosphatase inhibitors on ice. Equal quantities of the proteins (40 μg/well) were separated by SDS‐PAGE and transferred onto a polyvinylidene fluoride membrane (EMD Millipore). The membranes were incubated at 4°C overnight with primary antibodies against the following proteins: mTOR (1:1000, Abcam), p‐mTOR (Ser2448, 1:1000, Santa Cruz Biotechnology, Inc.), p‐Tau (Ser396, 1:500, Abcam), LC3 (1:500, Cell Signaling Technology), Beclin‐1 (1:1000, Abcam), P62 (1:1000, Cell Signaling), cleaved caspase‐3 (1:500, Cell Signaling), Bax (1:1000, Cell Signaling), and Bcl‐2 (Cell Signaling), as well as β‐actin (Cell Signaling), which was used as the internal reference *protein*. The membranes were then incubated with the corresponding secondary antibodies (1:500, Cell Signaling). After that, the blots were developed with an enhanced chemiluminescence reagent (Pierce) and detected by an X‐ray film (XBT‐1, Eastman Kodak Company). The band intensity was quantified by densitometric analysis using the Image J software (NIH). Relative protein expression levels were obtained by normalizing to β‐actin.

### 2.5 Immunohistochemistry staining

Rats were deeply anesthetized and perfused transcardially with 0.9% saline followed by 4% paraformaldehyde. Then, the brain tissue was rapidly removed and postfixed in 4% paraformaldehyde. The brains were dehydrated, paraffin embedded, and sectioned at a thickness of 5 μm. After dewaxing, the slides were incubated with Aβ_1‐42_ primary antibody (1:200, Cell Signaling) overnight at 4°C according to the manufacturer's instructions. Images were observed under a microscope (Leica) and analyzed with ImageJ software (BX50‐FLA). The number of Aβ_1‐42_‐positive neurons was measured in a square region of 100 × 100 μm^2^ under 200x magnification, and the optical density of each slice in the selected area was calculated.

### 2.6 Statistical analysis

The data were analyzed by SPSS 22.0 software (SPSS Inc.). All values are presented as the mean ±standard deviation (SD). The normality of the data distribution was analyzed by the Shapiro‐Wilk test. Comparisons among three or more experimental groups were performed with one‐way analysis of variance (ANOVA) followed by Bonferroni's post hoc test if the data were normally distributed; if the data were not normally distributed, the Kruskal‐Wallis test was performed as a nonparametric test. Two‐way repeated‐measures ANOVA and Bonferroni's post hoc tests were used to measure the escape latency in the MWM, with days as the repeated factor. Differences with a *p* < 0.05 were defined as statistically significant.

### 2.7 Ethical Statement

This study was approved by the Animal Care and Use Committee at Fujian Medical University (Fuzhou, China) and performed in accordance with the Guide for the Care and Use of Laboratory Animals of the National Research Council. All efforts were made to minimize animal suffering and the number of animals used in the study.

## 3 RESULTS

### 3.1 Surgical trauma induced cognitive impairments in diabetic rats

**FIGURE 1:**
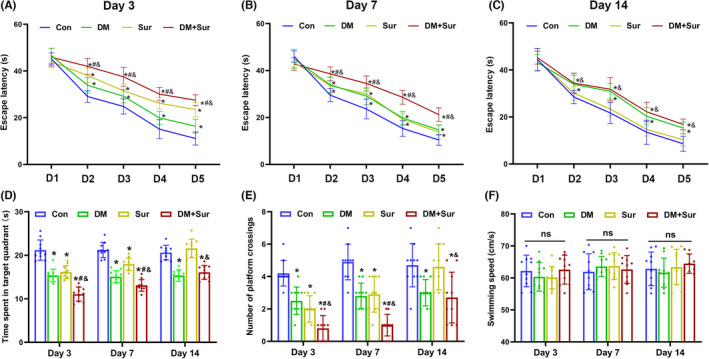
Surgical trauma induced neurocognitive dysfunction in diabetic rats. In the MWM test, the escape latency to reach the hidden platform during the place navigation test on days 3, 7, and 14 after surgery (A‐C). Time spent in the target quadrant during the spatial probe test (D). Number of platform crossings during the spatial probe test (E). Average swimming speeds of different groups during the spatial probe test (F). Data are expressed as the mean ±SD (*n* = 10 per group). ^∗^ *p* < 0.05 vs. the Con group; ^#^ *p* < 0.05 vs. the DM group; ^&^ *p* < 0.05 vs. the Sur group. Con: control group; DM: diabetes mellitus group; Sur: surgery group; DM+Sur: diabetes mellitus+Surgery group

We first evaluated the effects of surgical trauma on spatial learning and memory ability in diabetic rats using the MWM test on days 3, 7, and 14 after surgery. As shown in Figure 1, rats in the Sur group, DM group, and DM+Sur group displayed cognitive impairment, as indicated by longer escape latency (Figure 1A‐C, *p *< 0.05), less time spent in the target quadrant, and fewer platform crossings (Figure 1D‐E, *p *< 0.05), than the rats in the control group on days 3 and 7 after surgery. In addition, the DM+Sur group showed significantly more severe cognitive impairment than the Sur group or DM group (*p* < 0.05). Importantly, we found that the Sur group recovered on postoperative day 14, while cognitive impairment persisted in the DM+Sur group, indicating that surgical trauma induced serious and persistent neurocognitive disorders in diabetic rats. Moreover, there was no difference in average swimming speed among the four groups during the probe tests (Figure 1F), which suggested that the difference in behavioral outcomes in the MWM test was due to changes in cognitive function rather than changes in locomotor function or limb flexibility.

### 3.2 Surgical trauma induced hippocampal mTOR activation and autophagy inhibition in diabetic rats

**FIGURE 2:**
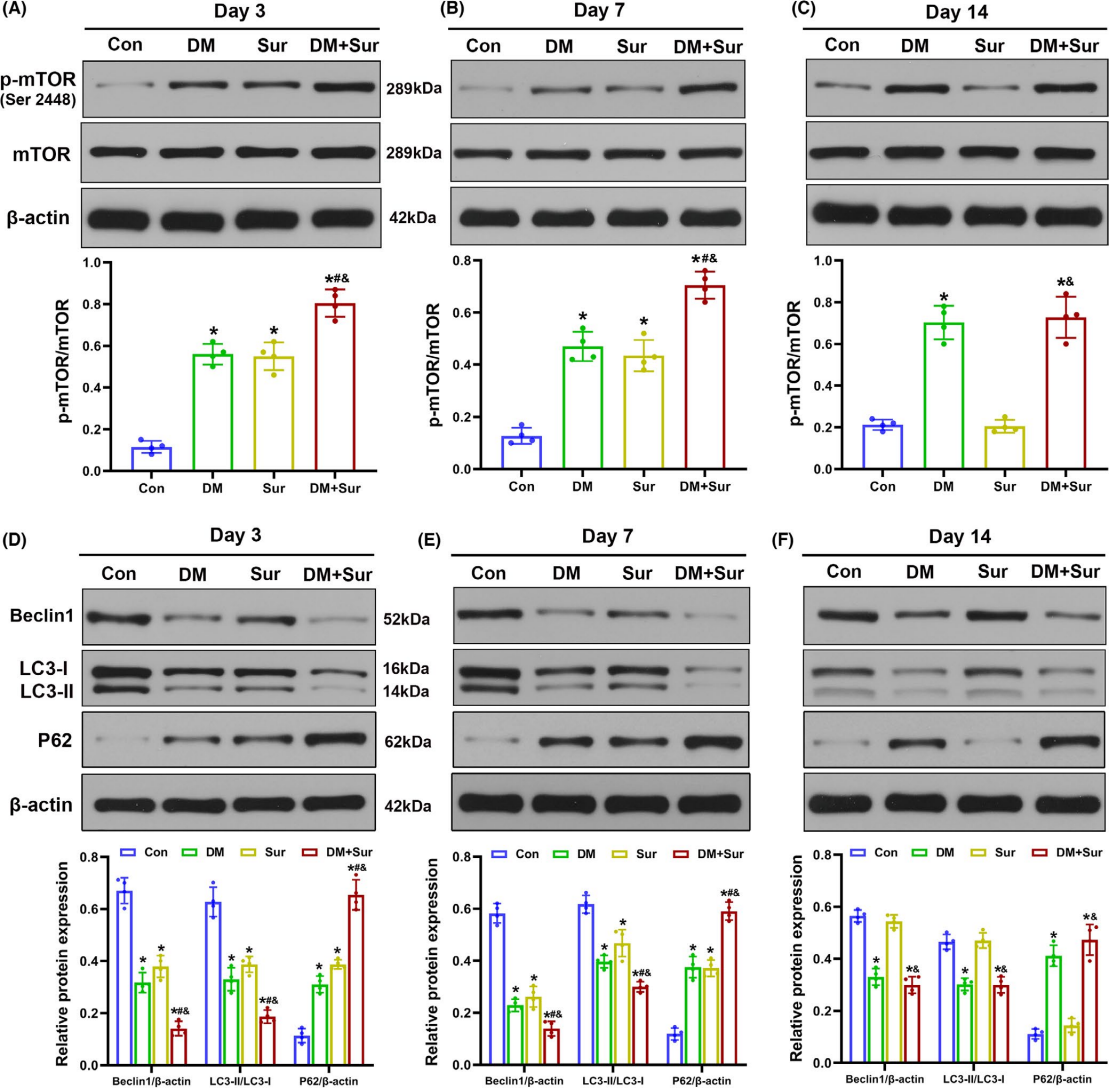
Surgical trauma induced hyperactivation of mTOR and inhibition of autophagy in the hippocampus of diabetic rats. Representative Western blot image and quantitative analysis of the Western blot (bar chart) illustrating the levels of p‐mTOR (Ser2448) expression (A‐C), LC3‐II/LC3‐I ratio, and Beclin‐1 and P62 expression (D‐F) in the hippocampus on days 3, 7, and 14 after surgery. Quantification of LC3‐I conversion to LC3‐II protein expression presented as the LC3‐II/LC3‐I ratio. Data are expressed as the mean ± SD (*n* = 4 per group). ^∗∗^ *p* < 0.05 vs. the Con group; ^#^ *p* < 0.05 vs. the DM group; ^&^ *p* < 0.05 vs. the Sur group

To investigate the effects of surgical trauma on mTOR‐mediated autophagy signaling in diabetic rats, the expression levels of p‐mTOR and autophagy‐related proteins (P62, Beclin‐1, and LC3‐II/LC3‐I ratio) in the hippocampus were examined using Western blot on days 3, 7, and 14 after surgery. We observed that both surgical trauma and DM significantly increased the expression levels of p‐mTOR and P62 (Figure 2A,B,D,E, *p *< 0.05) and decreased Beclin‐1 expression and the LC3‐II/LC3‐I ratio (Figure 2D, E, *p *< 0.05) on days 3 and 7 after surgery compared with those in the control group. Of note, the changes in the expression levels of p‐mTOR and autophagy‐related proteins were greater in the DM+Sur group than in the DM or Sur groups at both time points (all *p* < 0.05). Moreover, the levels of p‐mTOR, P62 and Beclin‐1 and the LC3‐II/LC3‐I ratio returned to baseline in the Sur group but remained largely unchanged in the DM+Sur group by postoperative day 14 (Figure 2C,F). Together, these results indicated that surgical trauma enhanced mTOR activation and persistent autophagy impairment in diabetic rats.

### 3.3 Surgical trauma promoted hippocampal tau phosphorylation and Aβ deposition in diabetic rats

**FIGURE 3:**
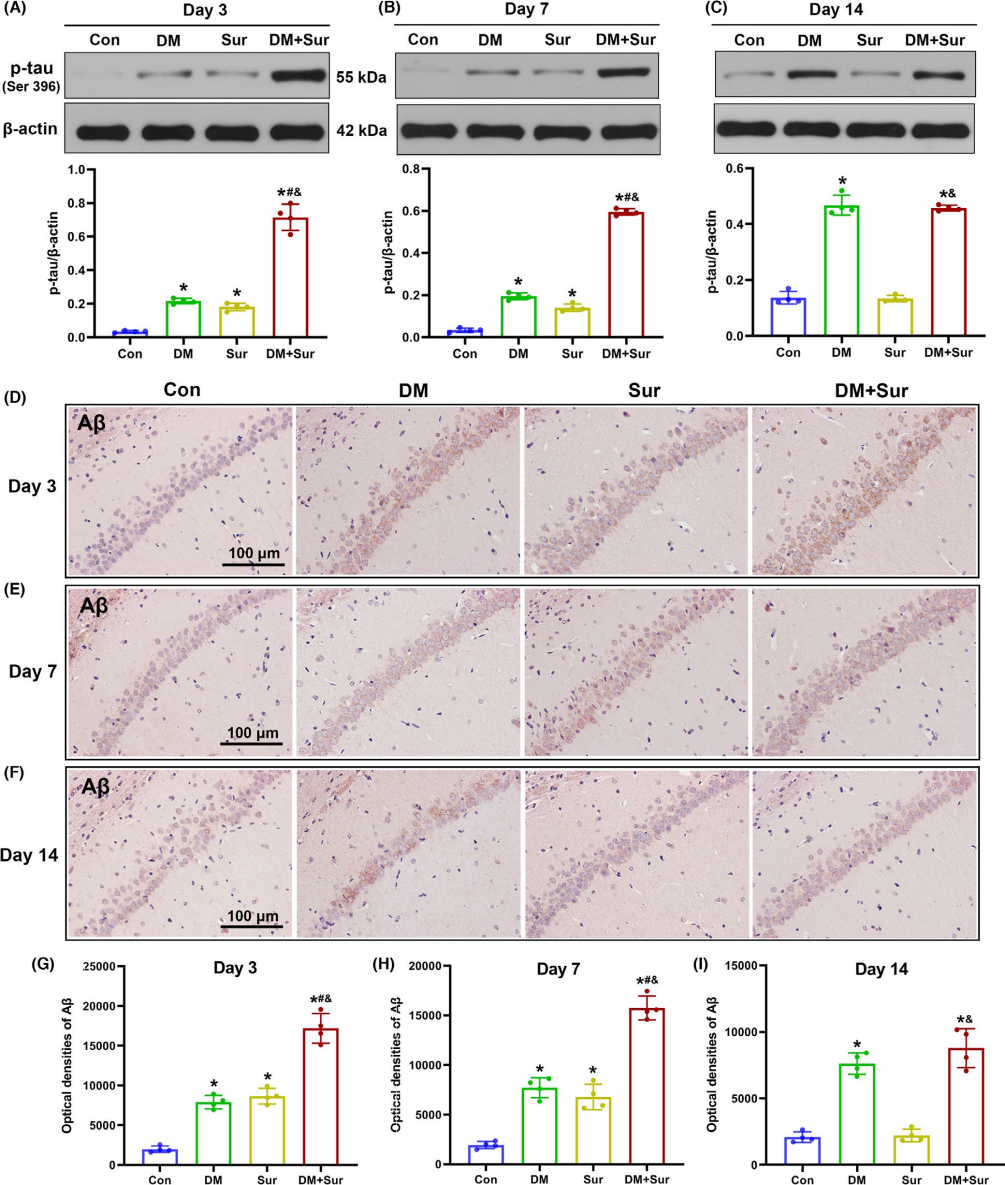
Surgical trauma promoted tau hyperphosphorylation and Aβ deposition in the hippocampus of diabetic rats. Representative Western blot image and quantitative analysis of the Western blot (bar chart) illustrating the expression levels of p‐tau (Ser 396) in the hippocampus on days 3, 7, and 14 after surgery (A‐C). Representative IHC image (200x) and quantitative analysis of the optical densities of Aβ1‐42‐positive neurons (bar chart) showing the expression of Aβ protein in the hippocampus on days 3, 7, and 14 after surgery (D‐I). Data are expressed as the mean ±SD (*n* = 4 per group). ^∗^ *p* < 0.05 vs. the Con group; ^#^ *p* < 0.05 vs. the Sur group; ^&^ *p* < 0.05 vs. the DM group

Our study further examined the levels of hippocampal p‐tau by Western blot and quantitative analysis of the densities of Aβ_1‐42_‐positive neurons by Immunohistochemistry staining (IHC) on postoperative days 3, 7, and 14. We found that the levels of p‐tau protein expression (Figure 3A,B, *p *< 0.05) and Aβ protein immunoreactivity (Figure 3D,E,G,H, *p *< 0.05) were significantly increased in the Sur group and DM group compared with the control group on postoperative days 3 and 7 (all *p* < 0.05) and were further increased in the DM + Sur group (*p* < 0.05). Consistent with the change trends of mTOR and autophagy, the levels of p‐tau (Figure 3C) and Aβ protein (Figure 3F,I) roughly returned to baseline in the Sur group but remained upregulated in the DM+Sur group by postoperative day 14. These data suggested that surgical trauma induced the aggregation of hyperphosphorylated tau protein and Aβ deposition in diabetic rats.

### 3.4 Rapamycin treatment inhibited abnormal mTOR activation and promoted autophagy activity in the hippocampus

**FIGURE 4:**
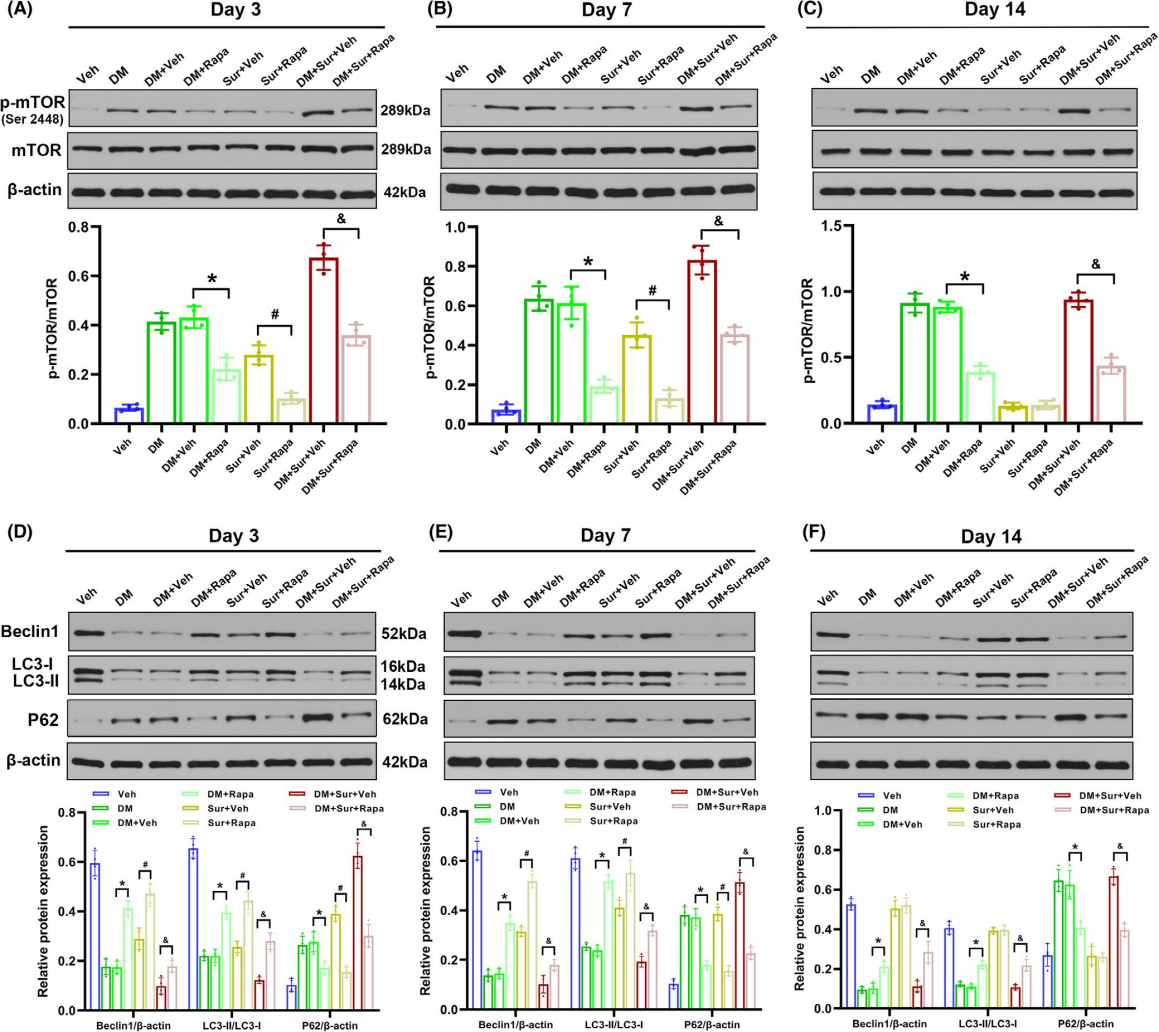
Rapamycin treatment inhibited the activation of mTOR and restored autophagy function in the hippocampus. Representative Western blot image and quantitative analysis illustrating the effects of rapamycin on the surgical trauma‐induced changes in hippocampal p‐mTOR (Ser 396) levels on days 3, 7, and 14 after surgery in diabetic rats (A‐C). Representative Western blot image and quantitative analysis illustrating the effects of rapamycin on the surgical trauma‐induced changes in hippocampal LC3‐II/LC3‐I ratio, Beclin‐1 and P62 levels on days 3, 7, and 14 after surgery in diabetic rats (D‐F). Data are mean ±SD (*n* = 4 per group). *p < 0.05, DM+Veh group vs. DM+Rapa group; ^#^ *p* < 0.05, Sur+Veh group vs. Sur+Rapa group; *^&^p* < 0.05, DM+Sur+Veh group vs. DM+Sur+Rapa group. Veh: vehicle group; DM: diabetes mellitus group; DM+Veh: diabetes mellitus group with vehicle treatment; DM+Rapa: diabetes mellitus group with rapamycin treatment; Sur+Veh: surgery group with vehicle treatment; Sur+Rapa: surgery group with rapamycin treatment; DM+Sur+Veh: diabetes mellitus+Surgery group with vehicle treatment. DM+Sur+Rapa: diabetes mellitus+Surgery group with rapamycin treatment

To investigate whether the hyperactivation of mTOR was involved in surgery‐induced autophagy impairment in diabetic rats, we then evaluated the effects of rapamycin on the expression levels of P62 and Beclin‐1 and the ratio of LC3‐II/LC3‐I in the hippocampus. Western blot analysis revealed that rapamycin treatment markedly reduced hippocampal p‐mTOR and P62 expression (Figure 4, *p* < 0.05) while increasing Beclin‐1 expression and the LC3‐II/LC3‐I ratio (Figure 4D‐F, *p *< 0.05) on days 3, 7, and 14 after surgery compared with those in the Sur group, DM group, and DM+Sur group. These data suggested that inhibition of mTOR by rapamycin pretreatment could effectively reverse mTOR hyperphosphorylation and restore autophagy impairment induced by surgery in diabetic rats.

### 3.5 Rapamycin treatment attenuated tau hyperphosphorylation and Aβ deposition in the hippocampus

**FIGURE 5:**
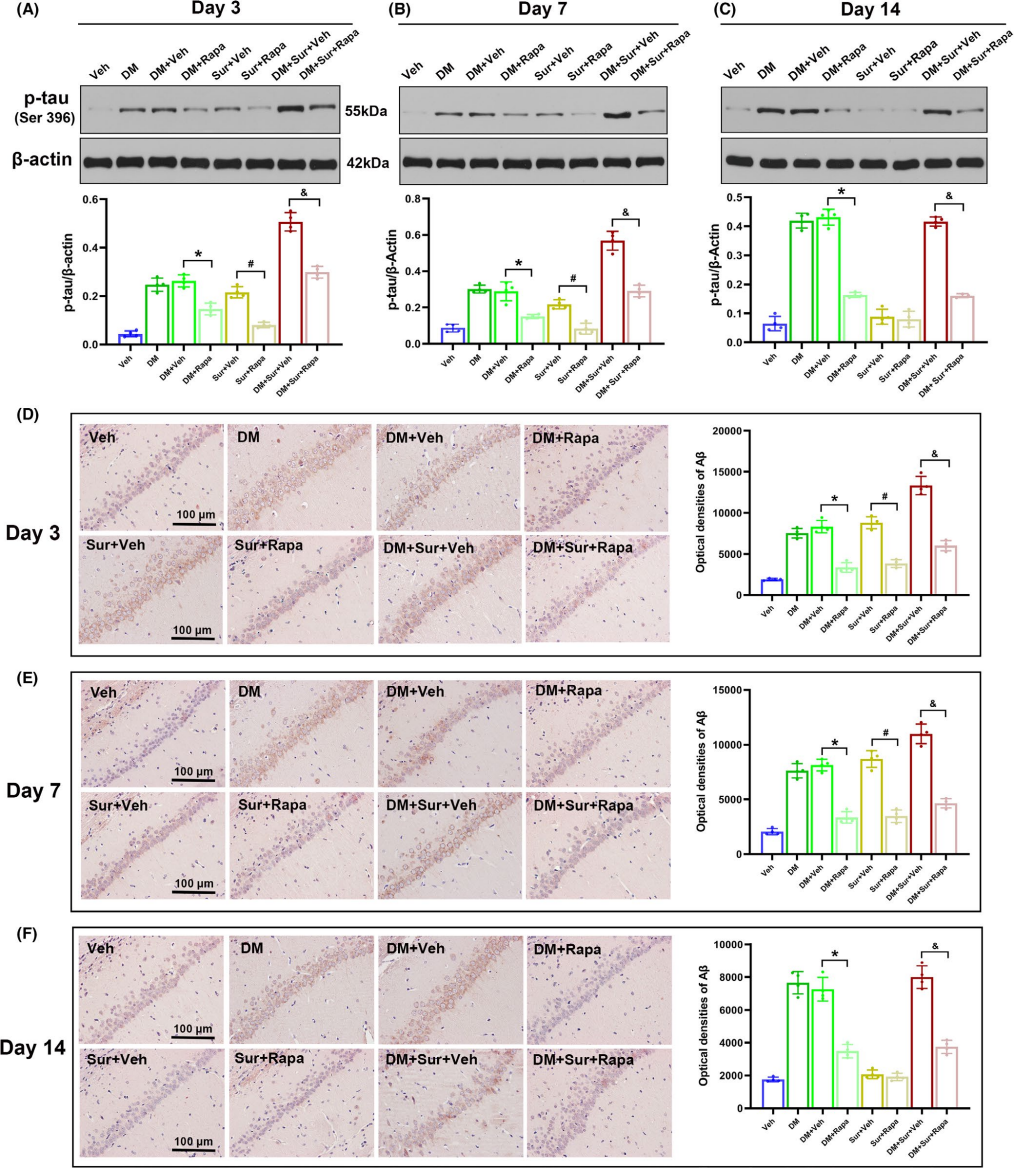
Rapamycin treatment inhibited the phosphorylation of tau protein and attenuated the deposition of Aβ in the hippocampus. Representative Western blot image and quantitative analysis illustrating the effects of rapamycin on the surgical trauma‐induced changes in hippocampal p‐tau (ser 396) levels on days 3, 7, and 14 after surgery in diabetic rats (A‐C). Representative IHC image and quantitative analysis of the optical densities of Aβ1‐42‐positive neurons (bar chart) showing the effects of rapamycin on the surgical trauma‐induced changes in hippocampal Aβ protein on days 3, 7, and 14 after surgery in diabetic rats (D‐F). Data are expressed as the mean ± SD (*n* = 4 per group). *p < 0.05, DM+Veh group vs. DM+Rapa group; ^#^ *p* < 0.05, Sur+Veh group vs. Sur+Rapa group; *^&^p* < 0.05, DM+Sur+Veh group vs. DM+Sur+Rapa group

To investigate whether the hyperactivation of mTOR was involved in surgery‐induced abnormal tau phosphorylation and Aβ deposition in diabetic rats, we evaluated the effects of rapamycin on the expression levels of p‐tau and Aβ_1‐42_ in the hippocampus on postoperative days 3, 7, and 14. Using Western blot and IHC analysis, we found that rapamycin treatment markedly reduced the levels of p‐tau protein expression (Figure 5A‐C, *p *< 0.05) and Aβ protein immunoreactivity (Figure 5D‐F, *p *< 0.05) on days 3, 7, and 14 after surgery compared with those in the Sur group, DM group, and DM+Sur group. Rapamycin pretreatment effectively attenuated excessive tau phosphorylation and Aβ deposition induced by surgery in diabetic rats.

### 3.6 Rapamycin treatment attenuated neuronal apoptosis and cognitive impairments in the hippocampus

**FIGURE 6:**
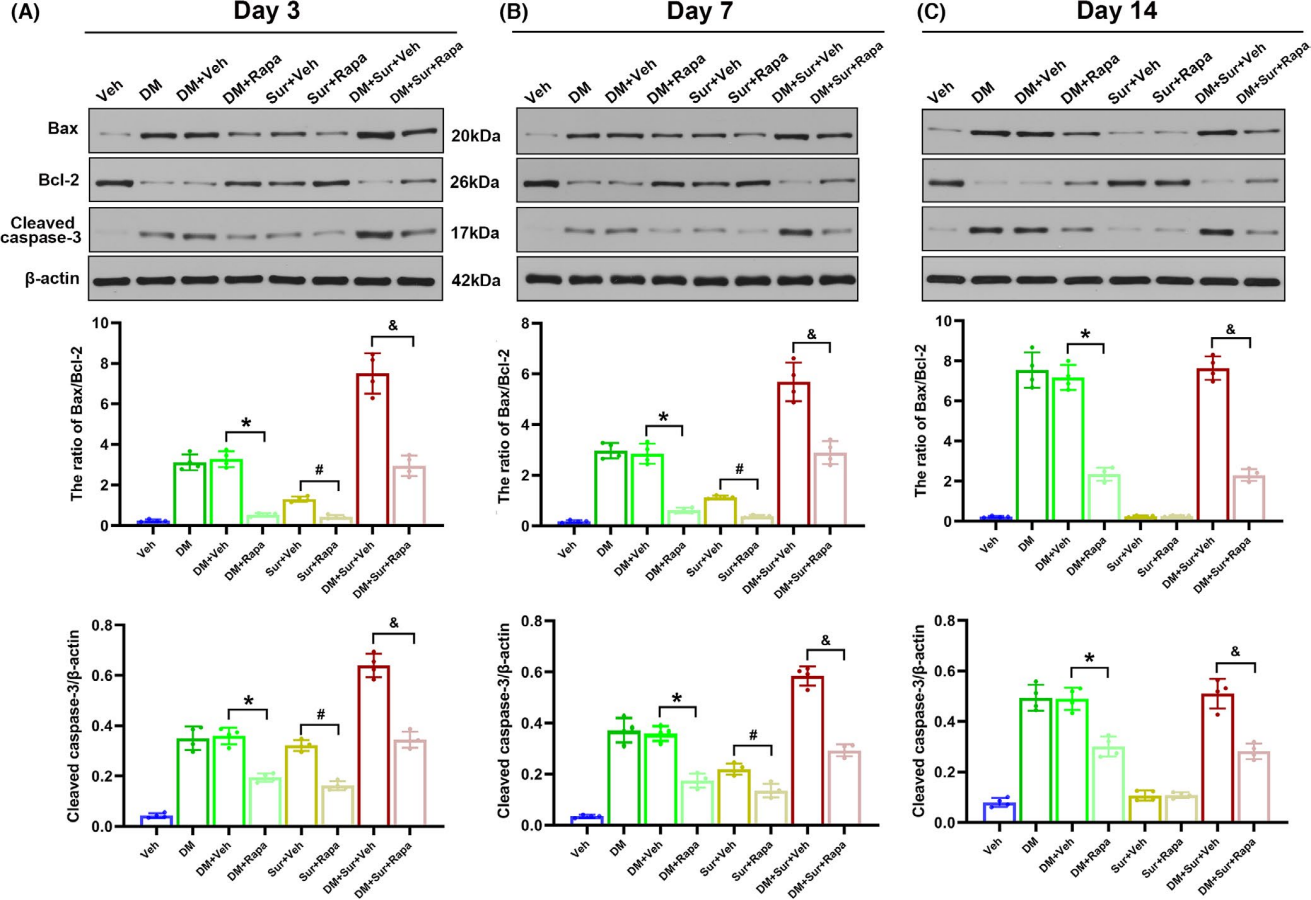
Rapamycin treatment prevented surgical trauma‐induced hippocampal apoptotic responses in diabetic rats. Representative Western blot image illustrating the effects of rapamycin on the surgical trauma‐induced changes in anti‐apoptotic protein Bcl‐2, pro‐apoptotic protein Bax, and cleaved caspase‐3 levels in the hippocampus of diabetic rats on days 3, 7, and 14 after surgery (A‐C). Quantification of Bax/Bcl‐2 was expressed as the ratio. Data are expressed as the mean ±SD (*n* = 4 per group). *p < 0.05, DM+Veh group vs. DM+Rapa group; ^#^ *p* < 0.05, Sur+Veh group vs. Sur+Rapa group; *^&^p* < 0.05, DM+Sur+Veh group vs. DM+Sur+Rapa group

Previous studies have proven that apoptosis is closely related to diabetes‐induced neuronal loss and neurocognitive impairment.^34^ Bax and caspase‐3 are known as crucial mediators of apoptosis, while Bcl‐2 is an anti‐apoptotic protein that inhibits cell apoptosis. Accordingly, to evaluate whether the hyperactivation of mTOR contributes to surgery‐induced neuronal apoptosis and cognitive dysfunction in diabetic rats, we evaluated the effects of rapamycin on the hippocampal Bax/Bcl‐2 ratio and cleaved caspase‐3 expression level, as well as on spatial learning and memory ability, using Western blot analysis and the MWM test. As shown in Figure 6, Western blot analysis showed that the Bax/Bcl‐2 ratio and cleaved caspase‐3 expression were significantly increased in the Sur group and DM group compared with the control group on postoperative days 3 and 7 and were further increased in surgery‐treated rats with diabetes (Figure 6A,B, *p *< 0.05). The Bax/Bcl‐2 ratio and cleaved caspase‐3 returned to baseline in the Sur group but remained upregulated in the DM+Sur group by postoperative day 14 (Figure 6C). Importantly, we demonstrated that rapamycin treatment markedly reduced the ratio of Bax/Bcl‐2 (*p* < 0.05) and cleaved caspase‐3 expression (*p* < 0.05) in the hippocampus compared with that in the Sur group, DM group, and DM+Sur group on days 3, 7, and 14 after surgery.

**FIGURE 7:**
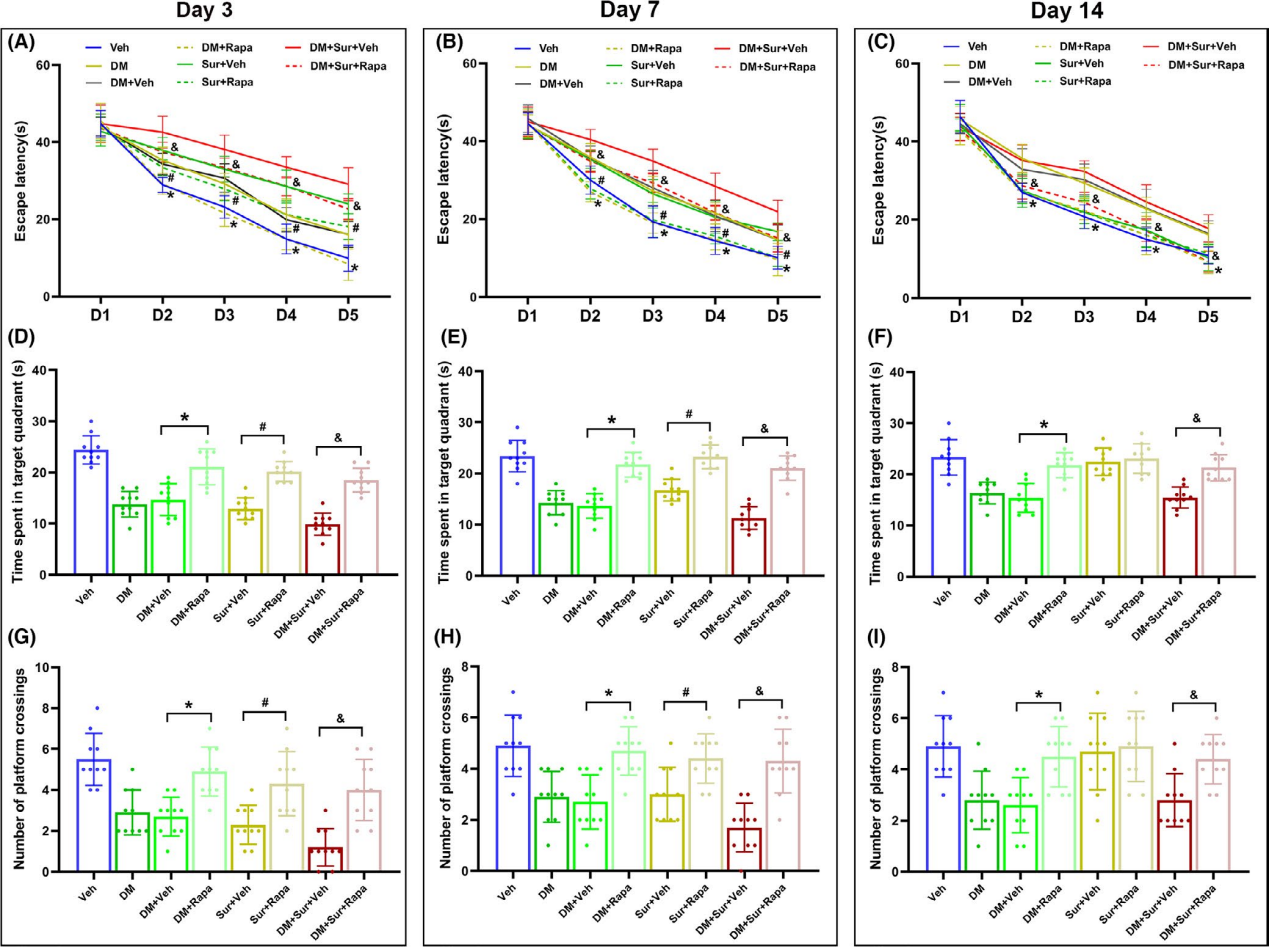
Rapamycin treatment alleviated surgical trauma‐induced cognitive impairment in diabetic rats. The escape latency to reach the hidden platform during the place navigation test on days 3, 7, and 14 after surgery (A‐C). Number of platform crossings during the spatial probe test (D‐F). Time spent in the target quadrant during the spatial probe test (G‐I). Data are expressed as the mean ± SD (*n* = 10 per group). *p < 0.05, DM+Veh group vs. DM+Rapa group; ^#^ *p* < 0.05, Sur+Veh group vs. Sur+Rapa group; *^&^p* < 0.05, DM+Sur+Veh group vs. DM+Sur+Rapa group

Similarly, the MWM results also showed that rapamycin was able to improve surgery‐induced cognitive impairment in the diabetic rats, as indicated by the decrease in escape latency (Figure 7A‐C, *p *< 0.05), the increase in time spent in the target quadrant (Figure 7D‐F, *p *< 0.05), and the increase in the number of platform crossings (Figure 7G‐I, *p *< 0.05) on days 3, 7, and 14 after surgery when compared with that in Sur group, DM group, and DM+Sur group. Collectively, these results suggested that the neuroprotective effect of rapamycin against surgery‐induced neuronal apoptosis and cognitive dysfunction in diabetic rats is strongly linked to the inhibition of mTOR hyperactivation.

## 4 DISCUSSION

Globally, the epidemic of diabetes presents a great challenge in the current healthcare system due to the rapidly increasing number of patients with diabetes scheduled for surgery.^5^ In the present study, surgical trauma was found to induce mTOR hyperactivation, abnormal tau phosphorylation, and Aβ deposition in the diabetic brain, which is associated with neuronal apoptosis and neurocognitive dysfunction. Furthermore, we found that inhibiting mTOR signaling and activating autophagy using the mTOR inhibitor rapamycin effectively attenuated hippocampal tau hyperphosphorylation, Aβ deposition, and apoptosis and improved neurocognitive dysfunction induced by surgery in diabetic rats. Mounting evidence has demonstrated that pathologic Aβ deposition, abnormal tau phosphorylation, and apoptosis are closely linked to cognitive impairments in various neurological and neurodegenerative diseases.^35^, ^36^, ^37^ Accordingly, our findings suggest that the modulation of mTOR‐mediated autophagy signaling could be a novel and promising strategy for the treatment of PND in diabetic patients.

PND are common neurological complications that include postoperative delirium (POD), postoperative cognitive decline (POCD), and delayed neurocognitive recovery and occur in 10% to 54% of patients after surgery.^2^ Although advanced age, neuroinflammation, and cerebrovascular dysfunction are thought to be important factors for cognitive impairment in the pathogenesis of neurodegenerative disorders,^38^, ^39^, ^40^, ^41^ the exact mechanisms underlying the pathogenesis of PND remain largely unclear. Recently, several clinical studies have indicated that diabetes and acute perioperative hyperglycemia are independent risk factors in the development of PND.^10^, ^12^ The hippocampus, a critical brain structure for memory and cognitive function, is particularly vulnerable to hyperglycemia damage in experimental diabetes models.^34^, ^36^ Therefore, in the present study, we chose diabetic rats to simulate the clinical situation of PND.

Tibial fractures with intramedullary fixation under isoflurane anesthesia are one of the most widely used animal models for PND studies.^32^ This model closely recapitulates features of clinical procedures such as hip or knee arthroplasty or fracture repair, which are often linked to neurological dysfunction in patients with underlying diseases. The MWM results in this study showed that surgical trauma caused transient learning and memory impairment that then recovered to normal levels by postoperative day 14 in nondiabetic rats, and these findings were consistent with a previous report.^25^ Furthermore, we found that neurocognitive dysfunction was exacerbated and persisted until postoperative day 14 in surgery‐treated rats with diabetes. Remarkable pathological changes characterize the brains of diabetic animals, particularly the hippocampus, and evidence has demonstrated that cognitive impairment in diabetes is closely associated with hippocampal neuronal apoptosis.^34^, ^42^ Similarly, our data showed that neuronal apoptosis in the hippocampus was increased in both the surgery‐treated and diabetic rats and was further increased in surgery‐treated rats with diabetes, as evidenced by the increased Bax/Bcl‐2 ratio and cleaved caspase‐3 expression compared with that in control rats. The level of neuronal apoptosis in the hippocampus returned nearly to normal in surgery‐treated rats by postoperative day 14 but remained high in surgery‐treated rats with diabetes. To our knowledge, the present study demonstrated for the first time that pretreatment with the mTOR inhibitor rapamycin ameliorated surgery‐induced hippocampal neuronal apoptosis and cognitive dysfunction in diabetic and nondiabetic rats. These data suggested that surgical trauma induced exaggerated and persistent neurocognitive disorders in diabetic rats, which were highly related to the hyperactivation of mTOR signaling.

mTOR is a protein kinase that regulates cell growth, proliferation, apoptosis, and autophagy via intracellular and extracellular signals.^13^ Numerous studies have revealed that dysfunction of mTOR signaling in the brain may be deeply correlated with cognitive dysfunction in DM and neurodegenerative diseases.^26^, ^27^, ^28^ Moreover, emerging evidence has proven that surgery or anesthetics can induce mTOR activation and autophagy impairment, contributing to cognitive dysfunction.^43^, ^44^ Autophagy is essential for the maintenance of cell homeostasis, as it clears damaged organelles and misfolded/toxic proteins, and autophagy dysfunction is involved in many neurodegenerative diseases.^18^, ^45^ P62, LC3, and Beclin‐1 are widely considered to be markers of autophagy. The LC3‐II/LC3‐I ratio and beclin‐1 levels directly correlate with autophagic activity, while the P62 levels inversely correlate with autophagy activity.^18^ In the present study, we showed that the levels of p‐mTOR and P62 increased, while the Beclin‐1 levels and the LC3‐II/LC3‐I ratio decreased in the hippocampus of rats following surgery. Notably, the changes in the p‐mTOR, P62, Beclin‐1, and LC3‐II/LC3‐I ratio were more obvious in surgery‐treated rats with diabetes than in surgery‐treated rats. Moreover, we found that rapamycin pretreatment effectively reversed surgical trauma‐induced mTOR hyperphosphorylation and promoted autophagy activity in diabetic rats. These molecular results were consistent with the behavioral findings, suggesting that mTOR‐inhibited autophagy activation in the hippocampus is involved in neurocognitive dysfunction induced by surgery and that DM exacerbates autophagy impairments in surgery‐treated rats due to hyperactivation of mTOR signaling in the hippocampus.

It has been reported that the blood concentration of rapamycin decreases to 4.7 nm at day 3 and 1 nM at day 7 after the last dose is given to mice.^46^ Finally, there is no doubt that rapamycin can be completely metabolized and removed from the body over time. In the present study, we observed sustained differences in p‐mTOR expression between the vehicle and rapamycin treatment groups as late as 14 days after surgery in diabetic rats. The reason for the consistent reduction in p‐mTOR expression at 14 days after the last dose of rapamycin administration is not clear, but it is likely because it still takes some time for the abnormal activation of the mTOR signaling pathway in the hippocampus even if rapamycin is completely metabolized by that time under diabetic conditions. Similarly, a recent study also reported that rapamycin treatment had a long‐lasting effect on reducing the expression of p‐mTOR and phospho‐s6 even up to 18 days in a rodent model of isoflurane exposure.^47^ Future studies are needed to investigate how long the pharmacologic effects of rapamycin can last after multiple consecutive days of injections.

Aβ and hyperphosphorylated tau are the main pathological hallmark proteins in Alzheimer's disease.^37^ A few studies also showed abnormal Aβ and p‐tau accumulation in the hippocampal neurons of experimental animal models or diabetic patients, which might induce oxidative stress, mitochondrial damage, and apoptosis, leading to diabetes‐associated cognitive impairment (DACI).^21^, ^48^, ^49^ mTOR signaling has been demonstrated to be involved in the deposition of Aβ protein and the abnormal phosphorylation of tau protein in neurodegenerative diseases, and inhibition of mTOR by rapamycin ameliorated Aβ and tau pathology and the associated cognitive deficits by enhancing the autophagic removal of damaged or toxic proteins.^48^, ^50^, ^51^ Accordingly, we proposed that the hyperactivation of mTOR may participate in tau hyperphosphorylation and Aβ deposition following surgery under diabetic conditions. Based on this, the present study further examined the levels of p‐tau and Aβ_1‐42_ in the hippocampus. We found that the levels of p‐tau and Aβ protein were increased in surgery‐treated rats and were further significantly increased in surgery‐treated rats with diabetes. Consistent with the change trends of mTOR and autophagy in our study, the p‐tau and Aβ levels roughly returned to normal in surgery‐treated rats by postoperative day 14 but remained elevated in surgery‐treated rats with diabetes. Importantly, the data showed that rapamycin treatment decreased the levels of tau phosphorylation at Thr396 and attenuated Aβ accumulation in the hippocampus after surgery. Collectively, these results suggested that mTOR‐mediated autophagy signaling may be responsible for neuronal apoptosis and neurocognitive dysfunction via abnormal tau hyperphosphorylation and Aβ deposition in the diabetic brain following anesthesia and surgery.

The present study does have limitations. First, our study focused on the alteration of cognition‐related protein expression in the hippocampus to reveal the potential mechanisms of PND in diabetic rats, but brain regions other than the hippocampus, such as the prefrontal cortex, might also be involved in cognitive impairment after surgery. Second, besides diabetes, aging is another risk factor for PND, and this deterioration is associated with increased neuroinflammation in the aged brain.^52^, ^53^ Neuroinflammation has been reported to implicate in autophagy dysfunction in neurodegenerative diseases. Neuroinflammation can lead to impairment of autophagy that exacerbates neurodegeneration, and conversely, a disruption of autophagy during pathological conditions can initiate or intensify neuroinflammation.^45^, ^54^, ^55^ Although the interaction between neuroinflammation and autophagy is complicated and controversial, this issue warrants further investigation in future work. Third, The MWM is a common and reliable test to assess hippocampal‐dependent spatial learning and memory in rodents undergoing tibial fracture surgery, some other behavioral experiments, such as the *Y*‐maze and fear conditioning tests, are needed to better evaluate the role of mTOR signaling in neurocognitive disorders.

## 5 CONCLUSIONS

In conclusion, the present study showed that surgical trauma induced exaggerated and persistent neurocognitive disorders in diabetic rats, probably via the hyperactivation of mTOR signaling. Importantly, we demonstrated that pretreatment with the mTOR inhibitor rapamycin ameliorated surgery‐induced neuronal apoptosis and cognitive impairment by enhancing autophagic removal of abnormal tau hyperphosphorylation and Aβ deposition in the hippocampus of diabetic rats. Our findings suggest that the modulation of mTOR signaling could be a promising therapeutic strategy for PND in patients with diabetes.

## CONFLICT OF INTEREST

The authors declare that they have no competing interests.

## AUTHOR CONTRIBUTIONS

Xiaohui Chen, Fei Gao, Cuicui Lin, and Xiaochun Zheng conceived and designed the experiments. Xiaohui Chen, Cuicui Lin, Andi Chen, Jianhui Deng, Pinzhong Chen, Mingxue Lin, and Bingxin Xie performed the experiments. Yanling Liao and Cansheng Gong analyzed the data. Xiaohui Chen, Fei Gao, and Xiaochun Zheng wrote, reviewed, and edited the article.

## Data Availability

All data generated or used during the study appear in the submitted article.
